# Modifiable risk factors of immediate and long-term outcomes in the operable and inoperable with left-sided infective endocarditis

**DOI:** 10.1016/j.heliyon.2024.e32041

**Published:** 2024-05-28

**Authors:** Jing-bin Huang, Chang-chao Lu, Zhao-ke Wen, Sheng-jing Liang

**Affiliations:** Department of Cardiothoracic Surgery, The People's Hospital of Guangxi Zhuang Autonomous Region, and Guangxi Academy of Medical Sciences, 6 Taoyuan Road, Nanning, 530021, Guangxi, China

**Keywords:** In-hospital mortality, Left-sided infective endocarditis, Inoperable, Operable, Serum creatinine 48 h post surgery

## Abstract

**Objectives:**

To evaluate the outcomes of left-sided infective endocarditis that can be operated on and cannot be operated on, and to focus on modifiable risk factors for immediate and long-term mortality.

**Methods:**

This study retrospectively investigated patients with left-sided infective endocarditis who occurred in our medical center between January 2006 and November 2022.

**Results:**

48 in-hospital deaths occurred (5.8 %, 48/832). We identified time from symptoms to admission and symptomatic neurological complications to be risk factors for multiple organ failure upon admission. Time from symptoms to admission and vegetation size in group of isolated medical treatment were significantly shorter than those in the group of heart operation. We also found that preoperative neurological complications, annulus destruction, levels of serum creatinine at 24 and 48 h post heart operation, and perivalvular leakage are risk factors for in-hospital mortality post heart operation. With 148 μmol/L as a cutoff level, the diagnostic sensitivity and specificity of serum creatinine level 48 h post surgery for in-hospital mortality post cardiac surgery are 100 % and 81.6 %, respectively. We found that vegetation size, ICU stay, postoperative serum creatinine at 48 h, left ventricular end diastolic size postoperative, and red blood cell transfusion were associated with all-time mortality.

**Conclusions:**

Early diagnosis and treatment, improvement of surgical techniques, good protection for heart, kidney and blood and close follow-up are advocated to conduce to better immediate and long-term outcomes of the operable and inoperable with left-sided infective endocarditis.

## 1 Introduction

Infective endocarditis (IE) refers to an infectious lesion in the heart and is a concerning disease. Although progress has been made in diagnosis and treatment, the rate of incidence and mortality of infective endocarditis have not decreased. Even in experienced centers, IE surgery still has the highest mortality rate among all valve diseases [[1], [2], [3]].

There is a difference between left and right infective endocarditis [4], and there is little research on the results of left infective endocarditis that cannot be operated on and can be operated on. We aim to evaluate the outcomes of left infective endocarditis that cannot be operated on and can be operated on, with a focus on the risk factors and intervention goals for immediate and long-term mortality in left-sided infective endocarditis.

We hypothesized that optimizing preoperative, perioperative, and postoperative factors could reduce the mortality and morbidity rate of left-sided infective endocarditis.

## 2 Patients in the study and methods

### 2.1 Design

We retrospectively investigated patients with left-sided infective endocarditis who underwent heart operation at our medical center between January 2006 and November 2022 and reviewed medical records.

### 2.2 Diagnosis

We diagnosed patients based on the improved Duke criteria [5] and reviewed surgical and pathological results to affirm diagnosis before surgery.

### 2.3 Criteria of eligibility

**Fig. 1:**
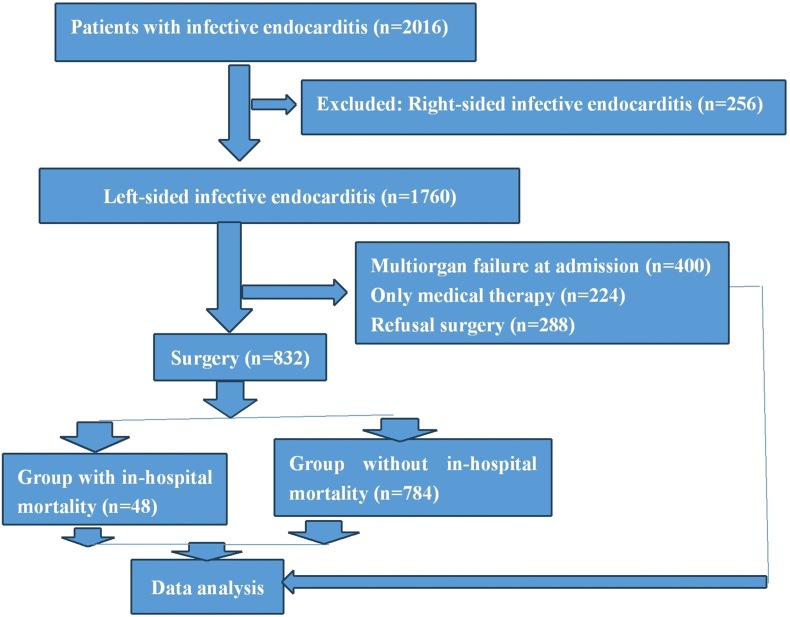
Flow chart of clinical trial.

Patients with left-sided infective endocarditis (≥18 years) during study period at our hospital were enrolled and patients with right-sided infective endocarditis and <18 years were excluded (Fig. 1).

### 2.4 Surgical technique

Heart operation was performed based on guidelines of AATS for the treatment of left-sided infective endocarditis [2,4]. All infected tissues and foreign material were debrided following by big irrigation. Allografts were the first choice for reconstruction of aortic root in patients with annulus destruction. Mitral valve repair was preferred, and replacements were completed with chordal preserved when possible. Operations included isolated mitral valve surgery, isolated aortic valve replacement, double valve operation, and Bentall + mitral valve replacement.

### 2.5 Peri- and post-operative treatment

Patients were all shifted to the intensive care unit post heart operation. Treatment of anticoagulation was routinely completed. Transthoracic echocardiograph was conducted in intensive care unit 1–7 days postoperatively.

### 2.6 Follow-up

All discharged survivors were followed up until the death date or the termination of the study. All patients undergo chest X-ray, electrocardiogram, and echocardiography examinations every 3–12 months at the outpatient department. We had an interview at the outpatient department with the patient directly or contacted by phone or WeChat during the last follow-up.

### 2.7 Variables analyzed

Variables in the Supplementary data were evaluated.

The time from symptoms to admission refers to the time between the onset of symptoms and the date of admission.

The time from symptoms to surgery refers to the time between the onset of symptoms and the date of surgery.

The in-hospital mortality rate refers to death within 30 days post operation or during the same hospitalization period.

### 2.8 Statistical analyses

We presented continuous variables as mean ± SE and performed normality tests on all variables using the Kolmogorov Smirnov test and used Kaplan Meier method to estimate survival rate. We also used the chi square test, Wilcoxon rank sum test or Kruskal Walls test (depending on the situation) assess the relationship between variables preoperative, intraoperative, and postoperative. We used contingency table method and logistic regression analysis to evaluate the relationship with perioperative risk factors. The correlation between two quantities is determined by the Spearman correlation coefficient. We draw the receiver operating characteristic (ROC) curve and its corresponding area under the curve (AUC) to assess the diagnostic value of risk and used the Youden index in ROC analysis to evaluate the optimal critical value. Kaplan Meier analysis was used to analyze survival rate, and the differences of survival rates between groups were tested using logarithmic rank test. We also used Multivariate Cox proportional hazard models. A P value less than 0.05 was statistically significant. All analyses were completed by IBM SPSS version 24.0 software (IBM SPSS Inc., USA).

## 3 Outcomes

### 3.1 Multiorgan failure upon admission in left-sided infective endocarditis

Age (46.75 ± 0.94 versus 44.57 ± 0.53 years, P = 0.022), vegetation size (11.79 ± 0.35 versus 10.74 ± 0.16 mm, P = 0.005), and serum creatinine upon admission (152.3 ± 10.56 versus 76.86 ± 0.84 μmol/L, P＜0.001 in group of multiorgan failure upon admission were significantly greater than those in group without multiorgan failure upon admission (Table 1).

**Table 1:** Multiorgan failure upon admission in left-sided infective endocarditis (n = 1760).

Variable	Group of multiorgan failure (n = 400)	Group of non-multiorgan failure (n = 1360)	P value	Total (n = 1760)
Male, n (%)	304 (76.0 %)	880 (69.6 %)	0.014	1184 (71.2 %)
Age, years	46.75 ± 0.94	44.57 ± 0.53	0.022	45.04 ± 0.37 (range, 18 to 82)
Weight, kg	56.69 ± 0.66	55.62 ± 0.34	0.167	55.82 ± 0.3 (range, 36 to 90)
Time from symptoms to admission, months	1.84 ± 0.09	2.28 ± 0.06	0.001	2.21 ± 0.05 (range, 0.1 to 12.3)
Vegetation size, mm	11.79 ± 0.35	10.74 ± 0.16	0.005	11.08 ± 0.15 (range, 0 to 29)
Aortic insufficiency, cm^2^	7.52 ± 0.52	3.95 ± 0.16	0.269	5.9 ± 0.2 (range,0 to 28)
Tricuspid insufficiency, cm^2^	4.60 ± 0.38	3.61 ± 0.12	0.745	3.8 ± 0.1 (range, 0 to 14.7)
Serum creatinine upon admission,μmol/L	152.3 ± 10.56	76.86 ± 0.84	＜0.001	82.3 ± 1.2 (range, 29 to 241)

Time from symptoms to admission (1.84 ± 0.09 versus 2.28 ± 0.06 months, P = 0.001) in group of multiorgan failure upon admission were significantly less than that in group without multiorgan failure (Table 1).

### 3.2 Risk factors of multiorgan failure upon admission (n = 400)

We identified male (OR: 1.382, 95 % CI: 1.067–1.790, P = 0.014), age (OR: 0.983, 95 % CI: 0.975–0.990, P＜0.001), and time from symptoms to admission (OR:1.127, 95 % CI: 1.052–1.208, P = 0.001), and symptomatic neurological complications (OR: 4.686, 95 % CI: 3.664–5.994, P＜0.001) to be risk factors of multiorgan failure upon admission by univariate analysis.

We also found male (OR: 1.521, 95 % CI: 1.154–2.005, P = 0.003), time from symptoms to admission (OR:1.672, 95 % CI: 1.235–3.568, ＜0.001), and symptomatic neurological complications (OR: 4.024, 95 % CI: 3.105–5.215, P＜0.001) to be risk factors of multiorgan failure upon admission using multivariate analysis (Table 2).

**Table 2:** Analysis of risk factors for multiorgan failure upon admission in left-sided infective endocarditis (n = 400).

Model	OR	95 % CI	P value
Univariate analysis			
Male	1.382	1.067–1.790	0.014
Age	0.983	0.975–0.990	＜0.001
Time from symptoms to admission	1.127	1.052–1.208	0.001
Symptomatic neurological complications	4.686	3.664–5.994	＜0.001
Multivariate analysis			
Male	1.521	1.154–2.005	0.003
Time from symptoms to admission	1.672	1.235–3.568	＜0.001
Symptomatic neurological complications	4.024	3.105–5.215	＜0.001

### 3.3 Isolated medical treatment and refusal operation in left-sided infective endocarditis (n = 1760)

224 patients were managed successfully by isolated medical treatment in left-sided infective endocarditis. Time from symptoms to admission (0.79 ± 0.05 versus 2.6 ± 0.1 months, P＜0.001) and vegetation size (5.0 ± 0.2 versus 12.0 ± 0.2 mm, P＜0.001) in group of isolated medical treatment were significantly less than both in group of heart operation (Table 3).

**Table 3:** Isolated medical treatment and refusal operation in left-sided infective endocarditis (n = 1760).

Variable	Group of isolated medical treatment (I) (n = 224)	Group of heart operation (II) (n = 832)	Group of refusal operation (III) (n = 288)	P value (I VS II)	P value (I VS III)	P value (II VS III)
Male, n	160 (71.4 %)	582 (70.0 %)	207 (71.9 %)	0.561	0.782	0.431
Age, years	42.9 ± 2.1	41.6 ± 3.1	52.3 ± 0.8	0.482	＜0.001	＜0.001
Body weight, kg	52.2 ± 1.2	55.1 ± 0.4	54.1 ± 0.5	0.579	0.004	0.231
Time from symptoms to admission, months	0.79 ± 0.05	2.6 ± 0.1	1.8 ± 0.1	＜0.001	＜0.001	＜0.001
Vegetation size, mm	5.0 ± 0.2	12.0 ± 0.2	11.1 ± 0.3	＜0.001	＜0.001	0.791

### 3.4 Preoperative and operative data

832 left-sided infective endocarditis patients who underwent heart operation were divided into group with in-hospital mortality (n = 48) and without in-hospital mortality (n = 784). There were 48 in-hospital deaths (5.8 %, 48/832) (Table 4).

**Table 4:** Preoperative and operative data in left-sided infective endocarditis (n = 832).

Variable	Group with in-hospital mortality (n = 48)	Group without in-hospital mortality (n = 784)	P value	Total (n = 832)
**Preoperative data**				
Male, n (%)	(%)	(%)		
Age, years	38.33 ± 0.72	39.29 ± 0.53	0.658	39.2 ± 0.5 (range, 8 to 71)
Weight, kg	52.33 ± 1.11	55.24 ± 0.44	0.109	55.1 ± 0.4 (range, 19 to 89)
Time from symptoms to admission，months	4.27 ± 0.43	2.44 ± 0.08	＜0.001	2.6 ± 0.1 (range, 0.1 to 12)
Vegetation size, mm	15.67 ± 0.48	10.69 ± 0.23	＜0.001	11.0 ± 0.2 (range, 0 to 28)
Preoperative left ventricular end diastolic dimension, mm	62.0 ± 1.1	62.46 ± 0.29	0.704	62.4 ± 0.3 (range, 52 to 84)
Preoperative left ventricular ejection fractions, %	69.33 ± 0.96	61.37 ± 0.28	＜0.001	61.8 ± 0.3 (range, 39 to 74)
Preoperative aortic insufficiency, cm^2^	7.00 ± 0.72	5.87 ± 0.25	0.269	5.9 ± 0.2 (range,0 to 28)
Preoperative mitral insufficiency, cm^2^	5.33 ± 0.22	7.98 ± 0.22	0.004	7.8 ± 0.2 (range, 0 to 19.5)
Preoperative tricuspid insufficiency, cm^2^	4.00 ± 0.41	3.82 ± 0.14	0.745	3.8 ± 0.1 (range, 0 to 14.7)
Serum creatinine before operation, μmol/L	160.3 ± 10.37	77.47 ± 0.84	＜0.001	82.3 ± 1.2 (range, 29 to 241)
**Operative data**				
Aortic cross-clamp time, minutes	115.7 ± 6.96	85.0 ± 1.22	＜0.001	86.8 ± 1.2 (range, 18 to 161)
CPB time, minutes	175.7 ± 7.8	140.3 ± 1.9	＜0.001	142.4 ± 1.8 (range, 54 to 282)
Mechanical ventilation time, hours	219.7 ± 11.7	40.9 ± 1.5	＜0.001	51.2 ± 2.1 (range, 2 to 333)
ICU stay, days	9.7 ± 0.5	4.7 ± 0.1	＜0.001	4.9 ± 0.1 (range, 2 to 14)
Hospitalized time postoperative, days	9.7 ± 0.5	20.0 ± 0.3	＜0.001	19.4 ± 0.3 (range, 7 to 50)
Serum creatinine 24 h after operation, μmol/L	151.7 ± 12.2	86.2 ± 1.3	＜0.001	90.0 ± 1.5 (range, 28 to 259)
Serum creatinine 48 h after operation, μmol/L	217.7 ± 12.5	97.6 ± 2.0	＜0.001	107.6 ± 2.4 (range, 26 to 351)
Fluid balance on operation day, ml	−233.3 ± 84.5	−602.2 ± 26.9	0.001	−581.0 ± 26.0 (range, −2500 to 900)
Fluid balance on 1st day postoperative, ml	−416.7 ± 113.7	−666.3 ± 39.3	＜0.001	−603.9 ± 38.6 (range, −6700 to 1500)
Fluid balance on 2nd day postoperative, ml	−33.3 ± 49.6	−579.6 ± 27.0	＜0.001	−548.1 ± 26.0 (range, −3200 to 1100)
Chest drainage, ml	753.3 ± 68.1	625.1 ± 14.5	0.036	633.1 ± 14.2 (range, 110 to 1720)
Postoperative left ventricular end diastolic dimension, mm	53.3 ± 0.25	48.2 ± 0.3	＜0.001	48.5 ± 0.2 (range, 30 to 64)
Postoperative left ventricular ejection fractions, %	57.0 ± 0.1	59.0 ± 0.3	0.087	58.6 ± 0.2 (range, 38 to 73)
Fresh-frozen plasma, ml	980.0 ± 60.8	619.6 ± 16.8	＜0.001	640.4 ± 16.5 (range, 0 to 2200)
Packed red blood cells, units	10.0 ± 0.9	2.4 ± 0.1	＜0.001	2.8 ± 0.1 (range, 0 to 18)

ICU, intensive care unit.

### 3.5 Preoperative data

Time from symptoms to surgery (4.27 ± 0.43 versus 2.44 ± 0.08 months, P＜0.001)，vegetation size (15.67 ± 0.48 versus 10.69 ± 0.23 mm, P＜0.001), preoperative left ventricular ejection fractions (69.33 ± 0.96 versus 61.37 ± 0.28 %, P＜0.001), and serum creatinine before operation (160.3 ± 10.37 versus 77.47 ± 0.84 μmol/L, P＜0.001) in group with in-hospital mortality were significantly greater than those in group without in-hospital mortality.

Preoperative mitral insufficiency (5.33 ± 0.22 versus 7.98 ± 0.22 cm^2^, P = 0.004) in group with in-hospital mortality was significantly less than that in group without in-hospital mortality (Table 4).

### 3.6 Operative data

Aortic cross-clamp time (115.7 ± 6.96 versus 85.0 ± 1.22 min, P＜0.001)，CPB time (175.7 ± 7.8 versus 140.3 ± 1.9 min, P＜0.001), mechanical ventilation time (219.7 ± 11.7 versus 40.9 ± 1.5 h, P＜0.001), ICU stay (9.7 ± 0.5 versus 4.7 ± 0.1 days, P＜0.001), serum creatinine 24 h post operation (151.7 ± 12.2 versus 86.2 ± 1.3 μmol/L, P＜0.001), serum creatinine 48 h post operation (217.7 ± 12.5 versus 97.6 ± 2.0 μmol/L, P＜0.001), chest drainage (753.3 ± 68.1 versus 625.1 ± 14.5 ml, P＜0.001), postoperative left ventricular end diastolic dimension (53.3 ± 0.25 versus 48.2 ± 0.3 mm, P＜0.001), fresh-frozen plasma (980.0 ± 60.8 versus 619.6 ± 16.8 ml, P＜0.001), and packed red cells (10.0 ± 0.9 versus 2.4 ± 0.1 units, P＜0.001) in group with in-hospital mortality were significantly greater than those in group without in-hospital mortality (Table 4).

### 3.7 Analysis of risk factors for in-hospital mortality after heart operation

We identified neurological complications before operation (OR: 3.583, 95 % CI: 1.895–6.775, P＜0.001), destruction of the annulus (OR: 5.625, 95 % CI: 2.930–10.80, P＜0.001), serum creatinine 24 h post operation (OR: 1.365, 95 % CI: 1.137–2.876, P＜0.001), serum creatinine 48 h post operation (OR: 1.562, 95 % CI: 1.256–3.659, P＜0.001), and paravalvular leak (OR: 12.50, 95 % CI: 8.719–17.199, P＜0.001) to be risk factors for in-hospital mortality after heart operation by univariate analysis. We also found neurological complications before operation (OR: 9.745, 95 % CI: 4.289–22.146, P＜0.001), destruction of the annulus (OR: 3.328, 95 % CI: 2.430–8.562, P＜0.001), serum creatinine 24 h post operation (OR: 1.476, 95 % CI: 1.273–3.693, P＜0.001), serum creatinine 48 h post operation (OR: 1.853, 95 % CI: 1.329–5.852, P＜0.001), and paravalvular leak (OR: 17.285, 95 % CI: 8.416–35.501, P＜0.001) to be risk factors for in-hospital mortality after heart operation by multivariate analysis (Table 5).

**Table 5:** Analysis of risk factors for in-hospital mortality after heart operation in left-sided infective endocarditis (n = 832).

Model	OR	95 % CI	P value
Univariate analysis			
Neurological complications before operation	3.583	1.895–6.775	＜0.001
Destruction of the annulus	5.625	2.930–10.80	＜0.001
Serum creatinine 24 h after operation	1.365	1.137–2.876	＜0.001
Serum creatinine 48 h after operation	1.562	1.256–3.659	＜0.001
Paravalvular leak	12.50	8.719–17.199	＜0.001
Multivariate analysis			
Neurological complications before operation	9.745	4.289–22.146	＜0.001
Destruction of the annulus	3.328	2.430–8.562	＜0.001
Serum creatinine 24 h after operation	1.476	1.273–3.693	＜0.001
Serum creatinine 48 h after operation	1.853	1.329–5.852	＜0.001
Paravalvular leak	17.285	8.416–35.501	＜0.001

Acute kidney injury (266/832, 32.0 %) and multiorgan failure (83/832, 10.0 %) were common early postoperative complications (Table 6).

**Table 6:** Operation and causes of in-hospital mortality and complications in left-sided infective endocarditis (n = 832).

Variable	Value
Operation	
Isolated aortic valve replacement, n	176 (21.2 %)
Isolated mitral valve surgery, n	368 (44.2 %)
Double valve operation, n	256 (30.8 %)
Bentall + MVR, n	16 (1.9 %)
ECMO, n	3 (0.4 %)
Causes of postoperative mortality	n (%)
Paravalvular leak + Cardiogenic shock + AKI + hepatic failure + septicemia	32 (3.8 %)
Intracerebral hemorrhage	16 (1.9 %)
Complications	
Acute kidney injury, n	257 (30.9 %)
Intubation time＞48 h	338 (40.6 %)
Hepatic failure, n	39 (4.7 %)
Respiratory failure, n	135 (16.2 %)
Ventricular fibrillation, n	33 (4.0 %)

AKI, acute kidney injury; MVR, mitral valve replacement; ECMO, Extracorporeal Membrane Oxygenation.

### 3.8 Association of serum creatinine 48 h post operation with in-hospital mortality after heart operation

**Fig. 2:**
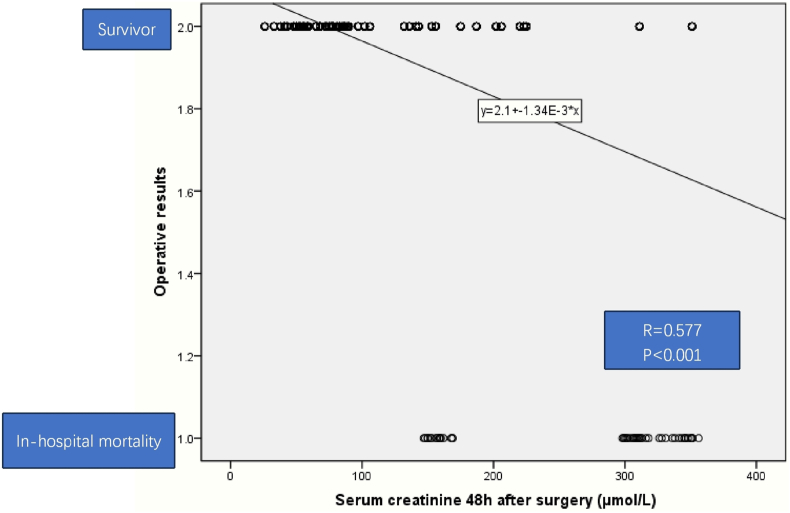
Association of serum creatinine 48 h after operation with in-hospital mortality after heart operation in left-sided infective endocarditis. There was a positive correlation between serum creatinine 48 h after operation and in-hospital mortality after heart operation in left-sided infective endocarditis (r = 0.577, P＜0.001) by Spearman correlations analysis.

**Fig. 3:**
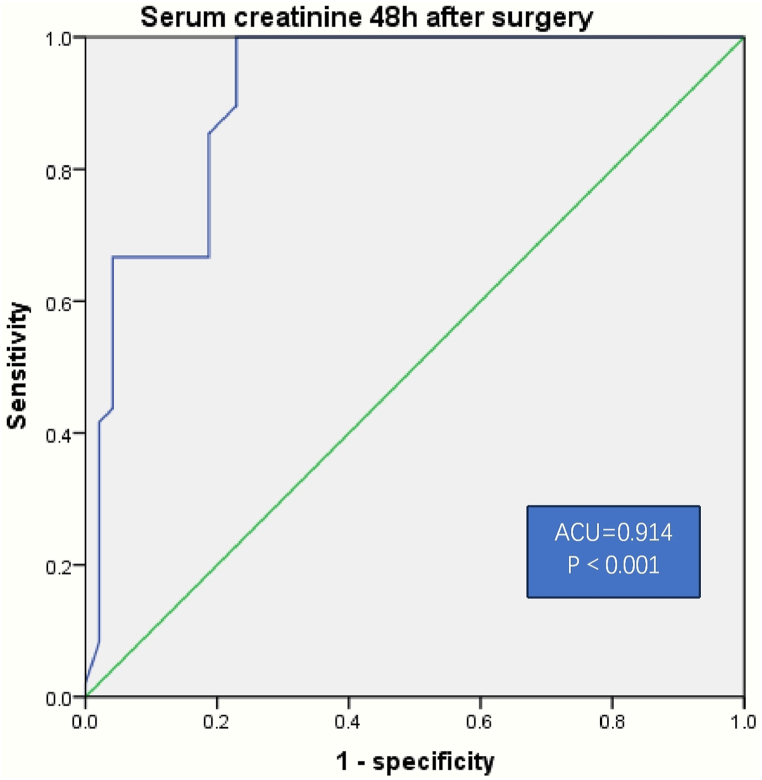
The ROC curve of diagnostic accuracy of serum creatinine 48 h after operation for predicting in-hospital mortality after heart operation in left-sided infective endocarditis. Using 148 μmol/L as a cutoff level, the value of serum creatinine 48 h after operation had 100 % sensitivity and 81.6 % specificity for diagnosis of in-hospital mortality after heart operation in left-sided infective endocarditis.

There was a positive correlation between serum creatinine 48 h post operation and in-hospital mortality after heart operation (r = 0.577, P＜0.001) by analysis of Spearman correlations (Fig. 2). The ROC curve of diagnostic accuracy of serum creatinine 48 h post operation was analyzed. Using 148 μmol/L as a cutoff level, the value of serum creatinine 48 h post operation had 100 % sensitivity and 81.6 % specificity for diagnosis of in-hospital mortality after heart operation. The AUC estimate for serum creatinine 48 h after surgery was 0.939 (P＜0.001), with a Youden index 0.816 (Fig. 3).

Comparison of groups of serum creatinine 48 h post operation < and ≥148 μmol/L was showed in Table 7.

**Table 7:** Comparison of groups of serum creatinine 48 h after operation < and ≥148 μmol/L (n = 832).

Variable	Group of serum creatinine 48 h after operation <148 μmol/L (n = 640)	Group of serum creatinine 48 h after operation ≥148 μmol/L (n = 192)	P value
Male, n (%)	400 (62.5 %)	112 (58.3 %)	0.148
Age, years	41.56 ± 0.45	43.67 ± 0.42	0.231
Weight s, kg	52.80 ± 0.42	62.67 ± 0.99	＜0.001
Time from symptoms to surgery，months	2.44 ± 0.10	2.90 ± 0.15	0.014
Vegetation size, mm	10.38 ± 0.24	12.98 ± 0.47	＜0.001
Preoperative LVEDD, mm	61.8 ± 0.31	64.33 ± 0.63	0.627
Preoperative LVEF, %	61.85 ± 0.31	61.83 ± 0.59	0.984
Preoperative aortic insufficiency, cm^2^	5.60 ± 0.27	7.04 ± 0.51	0.011
Preoperative mitral insufficiency, cm^2^	7.77 ± 0.26	8.01 ± 0.30	0.635
Preoperative tricuspid insufficiency, cm^2^	3.74 ± 0.15	4.14 ± 0.23	0.188
Serum creatinine before operation, μmol/L	72.03 ± 0.79	116.33±	＜0.001
In-hospital mortality, n	1 (0.2 %)	47 (24.5 %)	＜0.001
Aortic cross-clamp time, minutes	82.45 ± 1.37	101.17 ± 2.46	＜0.001
Cardiopulmonary bypass time, minutes	133.45 ± 2.12	172.08 ± 2.53	＜0.001
Mechanical ventilation time, hours	36.02 ± 1.48	101.83 ± 6.61	＜0.001
ICU stay, days	4.20 ± 0.09	7.42 ± 0.26	＜0.001
Hospitalized time postoperative, days	20.25 ± 0.31	16.75 ± 0.50	＜0.001
Serum creatinine 24 h after operation, μmol/L	74.28 ± 1.10	142.33 ± 3.48	＜0.001
Serum creatinine 48 h after operation, μmol/L	74.93 ± 1.15	216.67 ± 4.09	＜0.001
Fluid balance on operation day，ml	−749.5 ± 26.31	−19.17 ± 53.16	＜0.001
Fluid balance on 1st day postoperative，ml	−693.75 ± 45.03	−304.17 ± 33.9	＜0.001
Fluid balance on 2nd day postoperative，ml	−555.0 ± 26.93	−525.0 ± 67.95	0.627
Chest drainage，ml	610.0 ± 16.11	710.0 ± 29.54	0.003
Postoperative LVEDD, mm	48.3 ± 0.28	49.25 ± 0.41	＜0.001
Postoperative LVEF, %	59.1 ± 0.27	57.0 ± 0.4	＜0.001
Fresh-frozen plasma	609.0 ± 19.0	745.0 ± 32.0	0.004
Packed red blood cells	1.91 ± 0.07	5.79 ± 0.35	＜0.001

ICU, intensive care unit; LVEF, left ventricular ejection fractions; LVEDD, left ventricular end diastolic dimension.

### 3.9 Follow-up results

The total of 784 survivors were discharged and 750 (95.7 %, 750/784) were followed up to the termination date of the study or death date. The mean length of follow-up was 75.14 ± 1.80 months (range, 1 to 204). There were 87 deaths (87/750, 11.6 %) within 1 year after being discharged due to recurrence of IE and cerebral hemorrhage. The latest follow-up indicated that 639 survivors were in NYHA class I (639/663, 96.4 %) and 24 in class II (24/663, 3.6 %).

### 3.10 Risk factors for all-time mortality by Cox regression (n = 750)

**Fig. 4:**
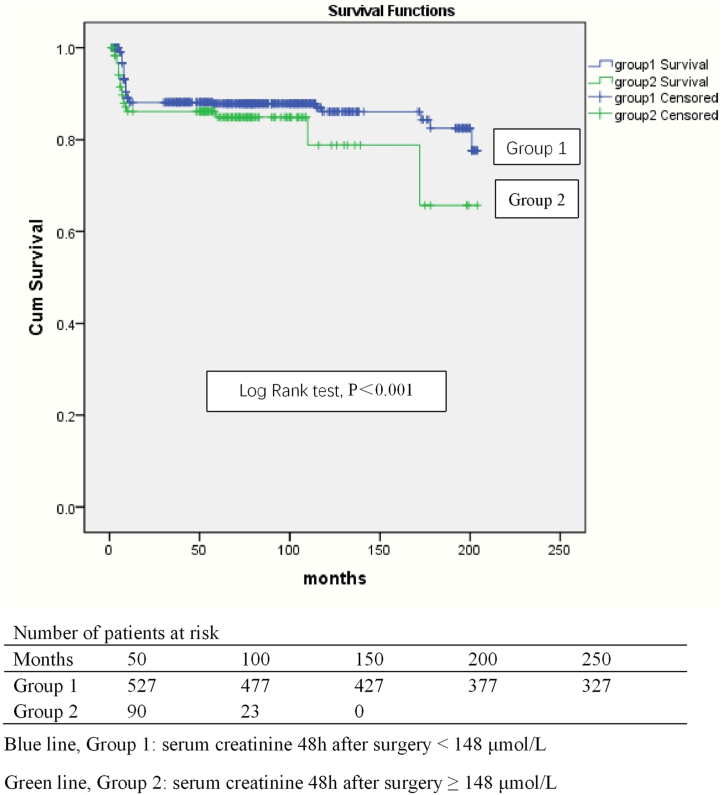
Kaplan-Meier curve for survival in patients with left-sided infective endocarditis stratified by the cutoff of serum creatinine 48 h after operation. Blue line, Group 1: serum creatinine 48 h after operation <148 μmol/L. Green line, Group 2: serum creatinine 48 h after operation ≥148 μmol/L. All-time mortality (0.2 % versus 24.5 %, P＜0.001) in group of serum creatinine 48 h after operation ≥148 μmol/L was significantly higher than that in group of serum creatinine 48 h after operation <148 μmol/L. (For interpretation of the references to colour in this figure legend, the reader is referred to the Web version of this article.)

We identified vegetation size (HR: 1.149, 95 % CI: 1.101–1.200, P＜0.001), ICU stay (HR: 1.960, 95 % CI: 1.732–2.217, P＜0.001), serum creatinine 48 h post operation (HR: 1.213, 95 % CI: 1.009–1.818, P＜0.001), postoperative left ventricular end diastolic dimension (HR: 1.232, 95 % CI: 1.169–1.299, P＜0.001), and packed red cells (HR: 1.647, 95 % CI: 1.453–2.687, P＜0.001) to be risk factors for all-time mortality by multivariate analysis of Cox regression (Table 8). Fig. 4 indicated different survival in left-sided infective endocarditis patients stratified by the cutoff value of serum creatinine 48 h post operation.

**Table 8:** Cox regression for all-time mortality (n = 750).

Model	HR	95 % CI	P value
Univariate analysis			
Vegetation size	1.185	1.056–1.531	＜0.001
ICU stay	1.443	1.364–1.526	＜0.001
Serum creatinine 48 h after operation	1.431	1.176–3.478	＜0.001
Postoperative left ventricular end diastolic dimension	1.143	1.108–1.398	＜0.001
Packed red blood cells	1.383	1.297–1.475	＜0.001
Multivariate analysis			
Vegetation size	1.149	1.101–1.200	＜0.001
ICU stay	1.960	1.732–2.217	＜0.001
Serum creatinine 48 h after operation	1.213	1.009–1.818	＜0.001
Postoperative left ventricular end diastolic dimension	1.232	1.169–1.299	＜0.001
Packed red blood cells	1.647	1.453–2.687	＜0.001

ICU, intensive care unit.

## 4 Discussion

Infectious endocarditis (IE) as a complicated disease, due to its long stay in hospital, high mortality (about 20–25 %) and high incidence rate, it has brought burden to the medical system. It is reported that half of patients underwent heart valve surgery. The average age of patients, healthcare related factors, artificial valves, coagulase negative Staphylococcus infection, and related complications increased [6]. Although the diagnosis and treatment programs have improved, the incidence rate of IE is still rising worldwide [[7], [8], [9], [10]]. In Europe in the twenty-first century, the median age is 63.7 years, with 69.4 % being male. Despite the increasing complexity of infective endocarditis cases, the prognosis has improved in recent years and the 6-month mortality rate has significantly decreased [11]. The prognosis of left-sided infective endocarditis is determined by multiple factors, including vegetation size. 21.1 % of patients experience new embolic events or die within 30 days. The cut-off value for predicting embolism events or 30-day mortality rate based on vegetation size is > 10 mm [12].

Our aims were to assess risk factors of immediate and long-term outcomes in the operable and inoperable with left-sided infective endocarditis. 48 in-hospital deaths occurred (5.8 %, 48/832). We identified time from symptoms to admission and symptomatic neurological complications to be risk factors for multiple organ failure upon admission. Time from symptoms to admission and vegetation size in group of isolated medical treatment were significantly shorter than those in the group of heart operation. We also found that preoperative neurological complications, annulus destruction, levels of serum creatinine at 24 and 48 h post heart operation, and perivalvular leakage are risk factors for in-hospital mortality post heart operation. With 148 μmol/L as a cutoff level, the diagnostic sensitivity and specificity of serum creatinine level 48 h post surgery for in-hospital mortality post cardiac surgery are 100 % and 81.6 %, respectively. We found that vegetation size, ICU stay, postoperative serum creatinine at 48 h, left ventricular end diastolic size postoperative, and red blood cell transfusion were associated with all-time mortality.

In our study, only 47.3 % (832/1760) left-sided infective endocarditis patients underwent surgery, and 22.7 % (400/1760) were excluded from heart operation due to multiorgan failure upon admission. Time from symptoms to admission is related to multiorgan failure upon admission. Symptomatic neurological complication is parameter of severity of disease. Only 12.7 % (224/1760) of left-sided infective endocarditis patients were treated successful with sole medical therapy, with less time from symptoms to admission and vegetation size compared to those undergoing heart operation. Timely diagnosis and treatment have significant implications for getting less time from symptoms to admission and vegetation size and early intervention of operation is important, because infective endocarditis is a complex, life-threatening disease. Completing a fast and precise diagnosis of infective endocarditis remains a grave challenge for us. The diagnostic value of repeated transthoracic echocardiography or transesophageal echocardiography for infective endocarditis decreases with increasing number of repetitions. In this case, the data cannot confirm that three or more transthoracic echocardiography or transesophageal echocardiography examinations are an effective strategy that can improve the diagnostic rate of all patients except for the selected suspected endocarditis patients [13].

Delaying diagnosis and starting treatment can result in complications and worse clinical results [14,15].

Socio-economic factors influence the clinical characteristics of infective endocarditis patients worldwide. Despite being young, patients from the poorest countries experience more frequent complications and higher mortality rates due to delayed diagnosis and lower surgical usage [16]. The timing of cardiac surgery after stroke in infective endocarditis is still controversial. There was no significant correlation between early surgery and increased in-hospital mortality. Delayed surgery for infective endocarditis patients after ischemic stroke does not have significant survival benefits. Further observational analysis, including detailed preoperative and postoperative clinical neurological findings and advanced imaging data (such as ischemic stroke size), may provide more precise recommendations for the optimal timing of valve surgery in infective endocarditis and recent stroke syndrome patients [17].

Due to the lack of a good primary, secondary, and tertiary prevention network, these patients seek medical treatment later in the hospital, usually diagnosed later and transferred to our tertiary hospital. A conceptual framework, including the required baseline information and requirements for implementing primary, secondary, and tertiary prevention, has been considered the best model for controlling infective endocarditis [18,19].

Patients with destructive annulus are prone to develop early paravalvular leak, a serious complication that significantly increases in-hospital mortality. Timely diagnosis and management can prevent the formation of periannular abscess and damage to the aortic valve annulus. Improvement of surgical techniques can decrease incidence of paravalvular leak. However, paravalvular leak in patients with IE and destructive annulus remain a grace challenge for heart surgeons [20,21]. More and more evidence suggest that aortic root replacement surgery is related to favorable rates of reoperation and early occurrence of perivalvular leakage, a hypothesis that is intuitive and supported by recognized surgical principles. During a one-year follow-up, patients who underwent aortic root replacement surgery had a lower risk of reoperation and the incidence of early perivalvular leakage. Aortic root replacement surgery can be the best practice choice for treating aortic valve endocarditis with periannular abscess and destructive aortic annulus. Aortic annulus destruction is related to early aortic valve leakage, in-hospital mortality, and 1-year mortality. Early paravalvular leak and sepsis were the main reasons of in-hospital mortality in IE. Early paravalvular leak occurred mainly in patients with destruction of the aortic annulus treated with 6.0 Prolene sutures and patch reconstruction. Aortic paravalvular leak generally do not occur in aortic root replacement (Bentall procedure). Early perivalvular leakage mainly occurs in patients with aortic annulus destruction, and 6.0 Prolene suture and patch reconstruction are used. Post aortic root replacement surgery (Bentall surgery), aortic valve leakage usually does not occur [22,23].

Patients with neurological complications before surgery are more severe, which partly explains why preoperative neurological complications are related to higher in-hospital mortality rates and longer mechanical ventilation time. A study showed that only 25.9 % of symptomatic patients with neurological complications require surgery upon admission, while the remaining patients (74.1 %) are prohibited from surgery due to death, severe septic shock, stroke and coma, or widespread neurological deficits. The prognosis of left-sided infective endocarditis is determined by multiple factors, including vegetation size. 21.1 % of patients experience new embolic events or die within 30 days. The cut-off value for predicting embolism events or 30-day mortality rate based on vegetation size is > 10 mm [24,25]. How to effectively diagnose and treat neurological complications remains a huge challenge we face [26,27].

We also identified a significant positive correlation between serum creatinine 48 h post operation and in-hospital mortality, and the cut-off value of serum creatinine 48 h post operation of 148 μmol/L with 100 % sensitive and 81.6 % specific for the diagnosis of in-hospital mortality. Serum creatinine 48 h post operation is a predictor and intervention target for decreasing in-hospital mortality. Effective measures should be taken to protect kidney function and make serum creatinine 48 h post operation less than 148 μmol/L. Serum creatinine 48 h post operation is a biomarker of immediate and long-term mortality of acute kidney injury postoperative in infective endocarditis. Optimizing pre-, peri-, postoperative factors that can reduce acute kidney injury leads to lower rates of acute kidney injury and immediate and long-term mortality [[28], [29], [30]].

The follow-up data of incidence rate and mortality showed that almost all deaths occurred within one year after operation, which meant that all discharged patients must be closely followed up. The size of vegetation, serum creatinine at 48 h post surgery, left ventricular end diastolic size, and packed red blood cells are associated with all-time mortality. Therefore, early diagnosis and treatment to avoid excessive vegetation size, as well as good protection of the kidneys, heart, and blood, are of great significance for long-term outcomes [31,32].

### 4.1 Perspectives

How to prevent multiple organ failure in patients with left infective endocarditis upon admission and provide optimal treatment remains a daunting challenge for us. The primary, secondary, and tertiary prevention, as well as early diagnosis and treatment networks for left-sided infective endocarditis, will contribute to better immediate and long-term outcomes.

### 4.2 Advantages and limitations of research

Our study is the first to elucidate the risk factors for immediate and long-term mortality in both the operable and inoperable with left-sided infective endocarditis, adding somethings value to current literature.

Due to the retrospective nature of the study and our hospital's role as a tertiary referral center, there may be selection bias. One more limitation is single center study. Well-designed multicenter prospective controlled studies, are needed, and projects aimed at reducing the incidence rate and mortality of hospitalization caused by left-sided infective endocarditis are encouraged.

## 5 Conclusions

Early diagnosis and treatment, improvement of surgical techniques, good protection for heart, kidney and blood and close follow-up are advocated to conduce to better immediate and long-term outcomes of the operable and inoperable with left-sided infective endocarditis.

## Ethics approval and consent to participate

The experimental protocol involving humans complies with the Helsinki Declaration and national guidelines, and has been approved by the Medical Ethics Committee of The People's Hospital of Guangxi Zhuang Autonomous Region (code: PHGX0186). They approved that authors do not need to obtain patient consent when publishing data related to patients in the study.

## Funding

This work was supported by the 10.13039/501100001809Natural Science Foundation of China (No: 81360014), the 10.13039/100012547Natural Science Foundation of Guangxi (No:2014GXNSFAA118234), the Guangxi key scientific and technological project (No: 2013BC26236), and the Projects in Guangxi Health Department (No: GZPT13-27).

## Availability of data and materials

The datasets used and/or analyzed during the current study are available from the corresponding author on reasonable request.

## CRediT authorship contribution statement

**Jing-bin Huang:** Writing – original draft, Funding acquisition, Formal analysis, Data curation, Conceptualization. **Chang-chao Lu:** Writing – review & editing, Resources, Methodology, Investigation. **Zhao-ke Wen:** Supervision, Resources, Methodology. **Sheng-jing Liang:** Visualization, Validation, Resources.

## Declaration of competing interest

The authors declare that they have no known competing financial interests or personal relationships that could have appeared to influence the work reported in this paper.

## References

[1] Cahill T.J., Baddour L.M., Habib G. (2017). Challenges in infective endocarditis. J. Am. Coll. Cardiol..

[2] Pettersson G.B., Hussain S.T. (2019). Current AATS guidelines on surgical treatment of infective endocarditis. Ann. Cardiothorac. Surg..

[3] Chu V.H., Park L.P., Athan E. (2015). International Collaboration on Endocarditis (ICE) Investigators*. Association between surgical indications, operative risk, and clinical outcome in infective endocarditis: a prospective study from the International Collaboration on Endocarditis. Circulation.

[4] Otto C.M., Nishimura R.A., Bonow R.O. (2020). ACC/AHA guideline for the management of patients with valvular heart disease: a report of the American College of Cardiology/American Heart Association Joint Committee on clinical practice guidelines. Circulation.

[5] Li J.S., Sexton D.J., Mick N. (2000). Proposed modifications to the Duke criteria for the diagnosis of infective endocarditis. Clin. Infect. Dis..

[6] Santos D.A.M., Siciliano R.F., Besen B.A.M.P. (2024). Changing trends in clinical characteristics and in-hospital mortality of patients with infective endocarditis over four decades. J Infect Public Health.

[7] Slipczuk L., Codolosa J.N., Davila C.D. (2013). Infective endocarditis epidemiology over five decades: a systematic review. PLoS One.

[8] Njuguna B., Gardner A., Karwa R. (2017). Infective endocarditis in low- and middle-income countries. Cardiol. Clin..

[9] Cahill T.J., Prendergast B.D. (2016). Infective endocarditis. Lancet.

[10] Huang J.B., Wen Z.K., Lu C.C. (2023). Risk factors of prolonged intensive care unit stay following cardiac surgery for infective endocarditis. Medicine (Baltim.).

[11] Ambrosioni J., Hernández-Meneses M., Durante-Mangoni E. (2023). International collaboration for endocarditis (ICE) investigators. Epidemiological changes and improvement in outcomes of infective endocarditis in Europe in the twenty-first century: an international collaboration on endocarditis (ICE) prospective cohort study (2000-2012). Infect. Dis. Ther..

[12] Sambola A., Lozano-Torres J., Boersma E., ESC EORP EURO-ENDO Registry Investigator Group (2023). Predictors of embolism and death in left-sided infective endocarditis: the European Society of Cardiology EURObservational research programme European infective endocarditis registry. Eur. Heart J..

[13] Vieira M.L., Grinberg M., Pomerantzeff P.M. (2004). Repeated echocardiographic examinations of patients with suspected infective endocarditis. Heart.

[14] Kang D.H., Kim Y.J., Kim S.H. (2012). Early surgery versus conventional treatment for infective endocarditis. N. Engl. J. Med..

[15] Thuny F., Beurtheret S., Mancini J. (2011). The timing of surgery influences mortality and morbidity in adults with severe complicated infective endocarditis: a propensity analysis. Eur. Heart J..

[16] Sengupta S.P., Prendergast B., Laroche C. (2022). Socioeconomic variations determine the clinical presentation, aetiology, and outcome of infective endocarditis: a prospective cohort study from the ESC-EORP EURO-ENDO (European Infective Endocarditis) registry. Eur Heart J Qual Care Clin Outcomes.

[17] Barsic B., Dickerman S., Krajinovic V. (2013). International Collaboration on Endocarditis–Prospective Cohort Study Investigators. Influence of the timing of cardiac surgery on the outcome of patients with infective endocarditis and stroke. Clin. Infect. Dis..

[18] Wyber R. (2013). A conceptual framework for comprehensive rheumatic heart disease control programs. Glob Heart.

[19] Mutagaywa R.K., Vroon J.C., Fundikira L., Wind A.M. (2022). Infective endocarditis in developing countries: an update. Front Cardiovasc Med.

[20] Anguera I., Miro J.M., Cabell C.H. (2005). ICE-MD investigators. Clinical characteristics and outcome of aortic endocarditis with periannular abscess in the International Collaboration on Endocarditis Merged Database. Am. J. Cardiol..

[21] Yang Bo, Caceres Juan, Farhat Linda (2021). Root abscess in the setting of infectious endocarditis: short and long-term outcomes. J. Thorac. Cardiovasc. Surg..

[22] Harris W.M., Sinha S., Caputo M. (2022). Surgical outcomes and optimal approach to treatment of aortic valve endocarditis with aortic root abscess. J. Card. Surg..

[23] Urbanski P.P., Lakew F., Dinstak W. (2018). Bentall procedure after previous aortic valve or complete root replacement: usefulness of self-assembled aortic valve conduit. J. Thorac. Cardiovasc. Surg..

[24] Huang J-b, Lu C-c, Wen Z-k (2023). Surgical treatment of left-sided infective endocarditis with symptomatic neurological complications before surgery in China. Front. Cardiovasc. Med..

[25] Sambola A., Lozano-Torres J., Boersma E., ESC EORP EURO-ENDO Registry Investigator Group (2023). Predictors of embolism and death in left-sided infective endocarditis: the European Society of Cardiology EURObservational research programme European infective endocarditis registry. Eur. Heart J..

[26] García-Cabrera E., Fernández-Hidalgo N., Almirante B. (2013). Neurological complications of infective endocarditis: risk factors, outcome, and impact of cardiac surgery: a multicenter observational study. Circulation.

[27] Kim R.W., Parikh C.R. (2011). Incidence, risk factors, and outcomes of acute kidney injury after pediatric cardiac surgery: a prospective multicenter study. Crit. Care Med..

[28] Ho J., Reslerova M., Gali B. (2012). Serum creatinine measurement immediately after cardiac surgery and prediction of acute kidney injury. Am. J. Kidney Dis..

[29] Husain-Syed F., Quattrone M.G., Ferrari F. (2020). Clinical and operative determinants of acute kidney injury after cardiac surgery. Cardiorenal Med..

[30] Ho J., Reslerova M., Gali B. (2012). Serum creatinine measurement immediately after cardiac surgery and prediction of acute kidney injury. Am. J. Kidney Dis..

[31] Huang J.B., Lu C.C., Wen Z.K. (2023). Impact of vegetation length on clinical complications during surgical intervention and long-term survival in infective endocarditis. Am. J. Cardiol..

[32] Njuguna B., Gardner A., Karwa R. (2017). Infective endocarditis in low- and middle-income countries. Cardiol. Clin..

