# Supplementary material for: Modifiable risk factors of immediate and long-term outcomes in the operable and inoperable with left-sided infective endocarditis
Source: Heliyon. 2024 May 28;10(11):e32041. doi: 10.1016/j.heliyon.2024.e32041 (PMC11177143; doi:10.1016/j.heliyon.2024.e32041)
Supplement: Multimedia component 1 [file mmc1.docx]

Supplementary data

Variables were evaluated including gender (female/male), age, weight, time between symptoms and surgery, NYHA class, rheumatic heart disease, valvular heart disease, coronary heart disease, left ventricular end diastolic dimension, left ventricular ejection fractions, aortic insufficiency, mitral insufficiency, tricuspid insufficiency, serum creatinine, mean intubation time, ICU retention time, hospitalized time after surgery, postoperative chest drainage, fresh-frozen plasma, packed red cells, fluid balance on operation day, the first day following operation and the second day following operation, acute renal injury, multiorgan failure, long-term intubation, hepatic failure, respiratory failure, ventricular fibrillation, use of inotropic medication, blood lactate, extracorporeal membrane oxygenation (ECMO) requirement, and death.
